# Case Report: A successful outcome of nadroparin calcium therapy for cerebral venous sinus thrombosis in a child with acute lymphoblastic leukemia

**DOI:** 10.3389/fped.2024.1448445

**Published:** 2024-09-10

**Authors:** Lichun Xie, Ye Xu, Guichi Zhou, Fen Chen, Changgang Li, Lian Ma, Feiqiu Wen

**Affiliations:** ^1^ Department of Pediatrics, The Third Affiliated Hospital of Guangzhou Medical University, Guangzhou, Guangdong, China; ^2^ Department of Hematology and Oncology, Shenzhen Children’s Hospital, Shenzhen, China

**Keywords:** cerebral venous sinus thrombosis, anticoagulation, nadroparin calcium, childhood acute lymphoblastic leukemia, pegylated-asparaginase

## Abstract

**Background:**

The appearance of cerebral venous sinus thrombosis (CVST) in childhood acute lymphocytic leukemia (ALL) is a rare life-threatening disease that can cause significant morbidity, neurological sequelae, and potentially poor outcomes.

**Case presentation:**

We present the case of a 13-year-old boy with ALL who developed CVST and intrinsic hemorrhage approximately 30 days after receiving chemotherapy with vincristine, dexamethasone, daunorubicin, and pegylated-asparaginase (PEG-Asp). He complained of a severe headache and then developed a generalized seizure at night. T1- and T2-weighted magnetic resonance imaging (MRI) and cerebral magnetic resonance venography sequences revealed superior sagittal sinus thrombosis and intrinsic hemorrhagic changes in the bilateral frontoparietal lobes. He received nadroparin calcium as the anticoagulant treatment and was switched to Erwinia asparaginase (Erwinia Asp) rather than PEG-Asp. Oxcarbazepine and clonazepam were started with good seizure control. Intrathecal treatment was delayed until 1 month later. Anticoagulation treatment was stopped for 24 h before and 6 h after lumbar puncture. Platelet transfusion was administered to ensure the platelet count remained at >50 × 10^9^/L. Oral acetazolamide (500–1,000 mg, daily) was administered to relieve headache and reduce intracranial pressure. Three months later, brain MRI showed a complete resolution of or significant improvement in the filling defect. Nadroparin calcium was administered for 1 week after switching to Erwinia Asp to prevent clot recurrence. He completed the 6-month chemotherapy and is doing well with no neurological sequelae and no recurrence of bleeding or thrombosis.

**Conclusions:**

Nadroparin calcium therapy appears to be safe and effective for pediatric CVST with ALL. The reintroduction of Erwinia Asp should be accompanied by anticoagulant therapy with nadroparin calcium**.**

## 1 Introduction

Acute lymphoblastic leukemia (ALL) is the most common childhood cancer. Venous thromboembolism (VTE) is a well-known complication of the treatment of pediatric ALL (1, 2). Cerebral venous sinus thrombosis (CVST) is a rare treatment-related but life-threatening complication of childhood ALL. The incidence of CVST in the population is 1%–2% in children treated for ALL and the mortality is approximately 10% (3). Because the symptoms and signs are non-characteristic and variable, early diagnosis of CVST is quite challenging. We present the case of a 13-year-old boy with parenchymal hemorrhage during induction chemotherapy.

## 2 Case report

**Figure 1:**
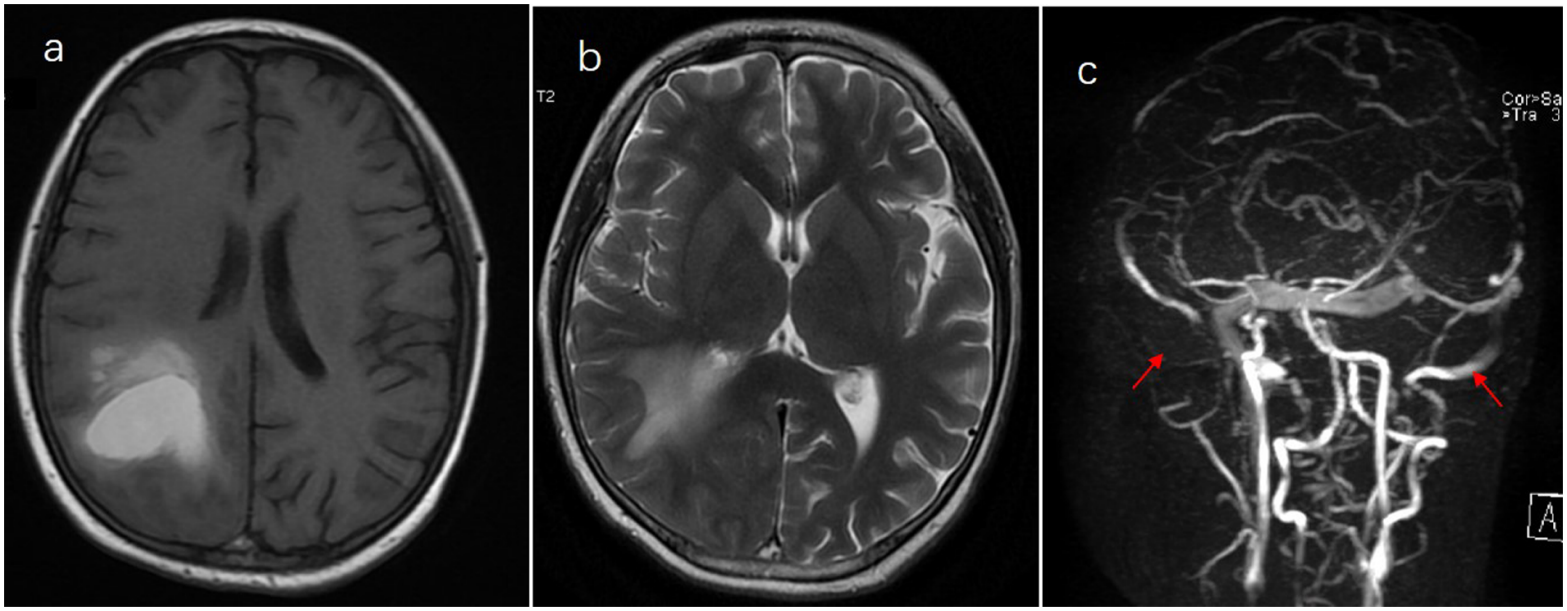
Thrombosis of the superior sagittal sinus and internal hemorrhage on the bilateral frontoparietal lobes. **(a)** T1. **(b)** T2+. **(c)** Cerebral magnetic resonance venography.

**Figure 2:**
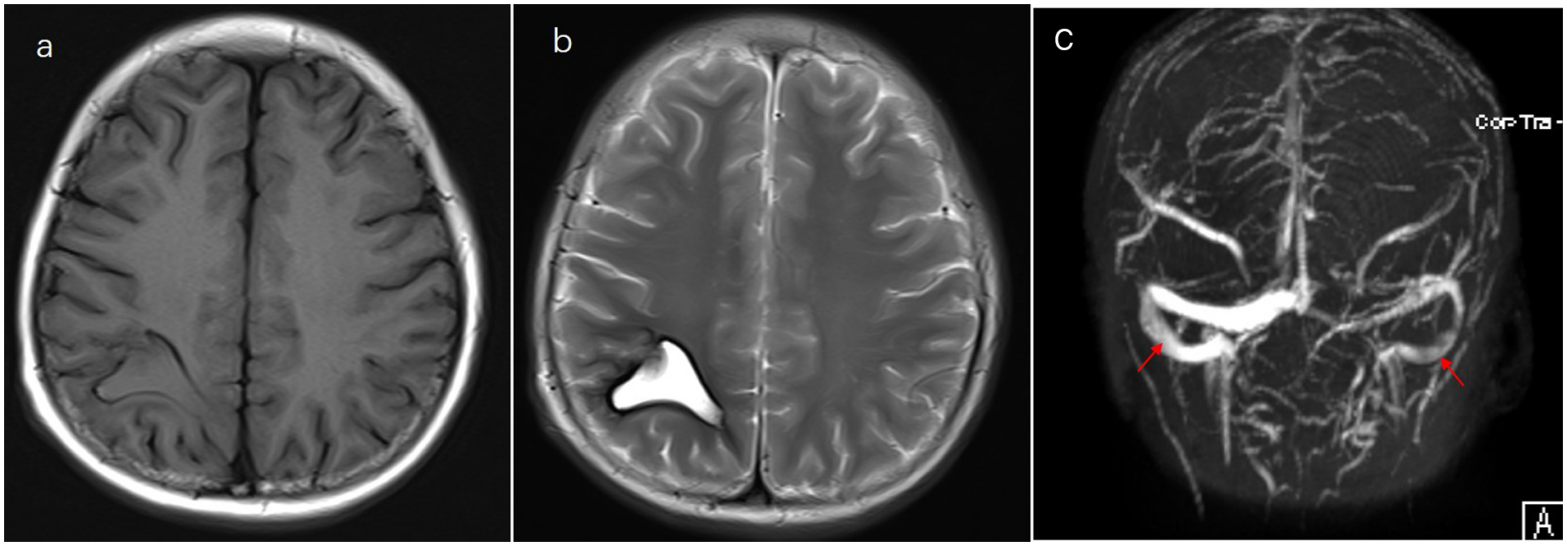
Evolution of changes on follow-up. **(a)** T1- and **(b)** T2-weighted MRI and **(c)** magnetic resonance venography imaging performed after the treatment.

A 13-year-old boy was diagnosed with B-cell immune phenotype ALL according to the South China Children's Leukemia Group (SCCLG-ALL) guidelines. Chromosomal translocations, intrachromosomal rearrangements, and genetic alterations were negative. Evidence of central nervous system (CNS) leukemia was negative. He was undergoing induction chemotherapy with vincristine, dexamethasone, daunorubicin, and pegylated-asparaginase (PEG-Asp) when he presented with a headache, and 30 days later, he developed a generalized seizure. Brain T_1_- and T_2_-weighted magnetic resonance imaging (MRI) and cerebral magnetic resonance venography revealed superior sagittal sinus thrombosis and intrinsic hemorrhagic alterations in the bilateral frontoparietal lobes (Figure 1). Anticonvulsants were administered. Hereditary thrombotic hemophilia was excluded. Plasma-infusion-corrected coagulation and fibrinogen level remained above 0.5 g/L. Platelet transfusion maintained a platelet count of >50 × 10^9^/L. Oxcarbazepine and clonazepam were used to control the seizures. Papilledema was ruled out. Two days later, his symptoms resolved slightly. One week later, brain MRI showed that intracranial hemorrhage had been effectively controlled, and chemotherapy for the SCCLG-ALL was reintroduced in combination with anticoagulation with nadroparin calcium. Nadroparin calcium treatment was started at a dose of 1 mg/kg twice daily subcutaneously. The second part of induction therapy included cyclophosphamide, mercaptopurine, and cytarabine. PEG-Asp was switched to Erwinia asparaginase (Erwinia Asp) in subsequent treatment phases. CNS leukemia was stratified into CNS-3 status. Intrathecal lumbar puncture (LP) was delayed for 1 month due to intracranial hemorrhage. The number of LPs with triple i.t. agents was modified to the CNS-3 status of the treatment protocol. The patient complained of headache, which is considered asymptomatic of chronic intracranial hypertension. Oral acetazolamide relieved the symptoms of chronic intracranial hypertension. Nadroparin calcium treatment was discontinued for 24 h before and 6 h after lumbar puncture. The nadroparin calcium dose was adjusted based on the platelet counts and antithrombin activity. Platelet transfusion was administered to ensure that the platelet count remained at >50 × 10^9^/L. Follow-up brain MRI at 3 months showed an improvement (Figure 2). Nadroparin calcium was adjusted to a once-a-day dose from that point. The total duration of nadroparin calcium treatment was extended to 1 week post-Erwinia Asp to prevent clot recurrence. No recurrence of bleeding or thrombosis occurred during treatment. The patient completed 6 months of chemotherapy and is doing well with no evidence of neurological deficit.

## 3 Discussion and literature review

CVST can present with various symptoms, including severe headache, focal neurological dysfunction (such as motor or sensory deficits), seizures, and altered consciousness (4). Early recognition of these symptoms is crucial for prompt diagnosis and treatment (5).

Idiculla et al. recommended that computed tomography (CT) and MR angiography should be the preferred diagnostic methods for CVST (6), as CT imaging without contrast agents has limitations and presents a false-negative rate of up to 40%. Additional sequences, such as diffusion-weighted imaging (DWI) and fluid-attenuated inversion recovery (FLAIR), can improve diagnostic accuracy (7).

Anticoagulant therapy is the mainstay of CVST treatment and should be started quickly (8, 9). Common anticoagulant options in pediatrics include unfractionated heparin (UFH), administered by continuous infusion, subcutaneous low molecular weight heparin (LMWH), and warfarin. In general, prophylactic LMWH does not significantly affect the activation of blood coagulation time (APTT), has minimal food interactions, and offers advantages due to its short half-life and flexibility in invasive surgical procedures (10). UFH is administered by continuous infusion. Nadroparin calcium can be administered subcutaneously at a dose of 86 IU/kg every 12 h. However, the pain associated with the subcutaneous nadroparin calcium administration and the need to involve parents in drug administration pose challenges. Nadroparin calcium eliminates the need for blood monitoring, making it more convenient. Warfarin, on the other hand, is available only in tablet form, which complicates its use in younger children. Furthermore, the prolonged duration of warfarin effects exhibits significant variability due to different factors. Concomitant use of sulfonamides, broad-spectrum antibiotics, and corticosteroids (commonly used in children with ALL) can affect the efficacy of warfarin, which requires close monitoring during clinical use (11). PREVAPIX-ALL was a phase III clinical trial in which no statistically significant treatment benefit was identified in pediatric ALL or lymphoma patients who received apixaban (12). The management of pegylated-asparaginase (PEG-Asp) re-administration after CVST in children with ALL during induction chemotherapy remains uncertain due to the lack of epidemiological studies. As ASP is a crucial drug in ALL treatment, there is controversy surrounding its association with venous thrombosis (13). In particular, Erwinia Asp, which has a shorter biological half-life than *Escherichia coli-*derived ASP and ASP-Peg, is recommended for patients with childhood ALL with CVST who need to return to ASP treatment after treatment suspension.

Children with ALL and extensive CVST can experience intracranial hypertension. This condition may be the result of damage to the arachnoid villi, which are present in the dural sinuses (14). The sagittal sinuses are particularly affected, leading to visual impairment, severe pain, difficulty concentrating, memory loss, and learning disabilities that can persist for months. To alleviate intracranial hypertension, oral acetazolamide therapy or repeated lumbar puncture therapy is recommended (15). In our case, oral acetazolamide was administered to relieve intracranial hypertension, along with oxcarbazepine and clonazepam to control epilepsy, resulting in a successful recovery.

## 4 Conclusion

We believe that the reintroduction of ASP treatment is safe with monitoring of antithrombin levels. Randomized controlled trials investigating the prevention and treatment of CVST will play a crucial role in determining effective clinical management.

## Data Availability

The original contributions presented in the study are included in the article/Supplementary Material, further inquiries can be directed to the corresponding author.

## References

[1] Gidl A Füreder A Benesch M Dworzak M Engstler G Jones N Incidence and risk factors of venous thromboembolism in childhood acute lymphoblastic leukaemia—a population-based analysis of the Austrian Berlin-Frankfurt-Münster (BFM) study group. Pediatr Hematol Oncol. (2023) 40(2):181–91. 10.1080/08880018.2022.2089791 35848787

[2] Athale UH Flamand Y Blonquist T Stevenson KE Spira M Asselin BL Predictors of thrombosis in children receiving therapy for acute lymphoblastic leukemia: results from Dana-Farber Cancer Institute ALL consortium trial 05-001. Pediatr Blood Cancer. (2022) 69(8):e29581. 10.1002/pbc.29581 35316569

[3] Caruso V Iacoviello L Di Castelnuovo A Storti S Mariani G Gaetano GD Thrombotic complications in childhood acute lymphoblastic leukemia: a meta-analysis of 17 prospective studies comprising 1752 pediatric patients. Blood. (2006) 108(7):2216–22. 10.1182/blood-2006-04-015511 16804111

[4] Ropper AH Klein JP. Cerebral venous thrombosis. N Engl J Med. (2021) 385(1):59–64. 10.1056/NEJMra2106545 34192432

[5] Ghanem KM Dhayni RM Al-Aridi C Tarek N Tamim H Chan AKC Cerebral sinus venous thrombosis during childhood acute lymphoblastic leukemia therapy: risk factors and management. Pediatr Blood Cancer. (2017) 64(12):e26694. 10.1002/pbc.26694 28660695

[6] Idiculla PS Gurala D Palanisamy M Vijayakumar R Dhandapani S Nagarajan E. Cerebral venous thrombosis: a comprehensive review. Eur Neurol. (2020) 83(4):369–79. 10.1159/000509802 32877892

[7] Idbaih A Boukobza M Crassard I Porcher R Bousser MG Chabriat H. MRI of clot in cerebral venous thrombosis: high diagnostic value of susceptibility-weighted images. Stroke. (2006) 37(4):991–5. 10.1161/01.STR.0000206282.85610 16484607

[8] Herishanu Y Misgav M Kirgner I Ben-Tal O Eldor A Naparstek E. Enoxaparin can be used safely in patients with severe thrombocytopenia due to intensive chemotherapy regimens. Leuk Lymphoma. (2004) 45(7):1407–11. 10.1080/10428190410001663671 15359641

[9] Liu TH Li XY Han JW Zhang YT Zhou DH Xu LH Acute lymphoblastic leukemia complicated with cerebral venous thrombosis in 14 children. Zhonghua Er Ke Za Zhi. (2020) 58(9):764–8. 10.3760/cma.j.cn112140-20200203-00058 32872718

[10] Goyal G Bhatt VR. L-asparaginase and venous thromboembolism in acute lymphocytic leukemia. Future Oncol. (2015) 11(17):2459–70. 10.2217/fon.15.114 26274336 PMC4976870

[11] Schulman S. How I treat recurrent venous thromboembolism in patients receiving anticoagulant therapy. Blood. (2017) 129(25):3285–93. 10.1182/blood-2017-03-742304 28483766

[12] O'Brien SH Rodriguez V Lew G Newburger JW Schultz CL Orgel E Apixaban versus no anticoagulation for the prevention of venous thromboembolism in children with newly diagnosed acute lymphoblastic leukaemia or lymphoma (PREVAPIX-ALL): a phase 3, open-label, randomised, controlled trial. Lancet Haematol. (2024) 11(1):e27–37. 10.1016/S2352-3026(23)00314-9 37980924

[13] Nowak-Göttl U Kenet G Mitchell LG. Thrombosis in childhood acute lymphoblastic leukaemia: epidemiology, aetiology, diagnosis, prevention and treatment. Best Pract Res Clin Haematol. (2009) 22(1):103–14. 10.1016/j.beha.2009.01.003 19285277

[14] Stam J. Thrombosis of the cerebral veins and sinuses. N Engl J Med. (2005) 352(17):1791–8. 10.1056/NEJMra042354 15858188

[15] Dlamini N Billinghurst L Kirkham FJ. Cerebral venous sinus (sinovenous) thrombosis in children. Neurosurg Clin N Am. (2010) 21(3):511–27. 10.1016/j.nec.2010.03.006 20561500 PMC2892748

